# The College of Nursing: Great Meeting at St. Thomas's Hospital: Full Verbatim Report

**Published:** 1916-04-15

**Authors:** 


### THE COLLEGE OF NURSING.

### A GREAT MEETING AT ST. THOMAS'S HOSPITAL.

### Full Verbatim Report.

A meeting of supporters of the College of Nursing was held in the Governors' Room, St. Thomas's Hospital, London, on Friday, April 7, 1916, the Hon. Arthur Stanley, M.P., M.V.O., in the chair. Amongst those present were:

**The Audience.**

Sir Cooper Perry, M.D., Miss Swift, R.R.C., Sir Henry Burdett, K.C.B., K.C.V.O., Dr. Jane Walker, M.D., Mr. Frank Debenham, Mr. C. Johnson, Miss Gibson.

Metropolitan Training Schools and Hospitals.

*Guy's Hospital*: Miss Haughton (matron), Miss Hogg (assistant matron), Miss Gratton (home sister), Miss Sheldon. *King's College Hospital*: Miss Ray, R.R.C. (matron). *London Hospital*: Mr. Ludovic Foster, Miss Monk (assistant matron). *Middlesex Hospital*: Mr. Comyns Berkeley, M.D., F.R.C.P., Mr. F. Cavendish Bentinck, Miss Montgomery (matron), Miss Habgood, Miss Thompson. *Royal Free Hospital*: Mr. J. Berry, F.R.C.S., Mr. R. Garratt (secretary), Miss Cox Davies, R.R.C. (matron), Mr. H. Phipson, Miss Tibbets, Miss Cooke. *St. Bartholomew's Hospital*: Lord Sandhurst, Dr. J. Calvert, Miss McIntosh (matron). *St. George's Hospital*: Mr. F. J. Frankau (deputy treasurer), Miss Cooper (matron). *St. Mary's Hospital*: Mr. T. Ryan (secretary), Miss Darbyshire (matron). *St. Thomas's Hospital*: Mr. T. Hall-Hall (Almoner), Dr. Box, F.R.C.P., Mr. E. Corner, F.R.C.S., Dr. Low, Dr. Turney, M.A., Mr. G. Q. Roberts, Miss Lloyd Still (matron). *University College Hospital*: Captain T. D. Butler, Mr. R. H. Horton Smith, K.C., Miss Finch (matron). *Westminster Hospital*: Mr. S. Quennell (secretary). *Bolingbroke Hospital*: Miss Foster (matron). *Brompton Hospital for Consumption*: Mr. Frederick Wood (secretary), Miss Rede (matron), Miss Barr. *City of London Hospital for Diseases of the Chest*: Miss Dalton (matron). *Great Northern Central Hospital*: Mr. Gilbert Panter (secretary), Miss Bird (matron). *National Hospital for the Paralysed and Epileptic*: Mr. G. Hamilton (secretary), Miss Spackman (matron). *Prince of Wales's General Hospital, Tottenham*: Mr. F. Drewett (director), Miss Bickerton (matron), Mr. J. Richardson. *Royal Hospital for Diseases of the Chest*: Miss Hamilton (acting matron). *Royal Hospital, Richmond*: Miss Dawson (matron). *West London Hospital*: Miss F. E. Nevile (matron). *No. 1 London General Hospital*: Miss Rundle (matron). *No. 3 London General Hospital*: Miss Holden, R.R.C. (matron). *Richmond Military Hospital*: Miss Fletcher.

Metropolitan Poor-Law Infirmaries.

*Chelsea Infirmary*: Miss Barton (matron). *City of Westminster Infirmary*: Miss Booth (matron). *Greenwich Infirmary*: Miss Ward (matron). *Kensington Infirmary*: Miss Alsop (matron). *Kingston Infirmary*: Miss Smith. *Mile End Military Hospital*: Miss Alder, Miss Shields. *Paddington Infirmary*: Miss Copeman (matron). *St. Marylebone Infirmary*: Miss Cockrell (matron). *Shoreditch Infirmary*: Mr. W. E. Fisher, F.R.C.S. *Whipp's Cross Infirmary, Leytonstone*: Miss Clark (matron). *Whitechapel Infirmary*: Miss Mowat (matron). *Woolwich Infirmary*: Miss Gooding (matron).

Provincial Training Schools, Hospitals, and Infirmaries.

*Ashton-under-Lyne Union Hospital*: Miss Seymour Yapp. *Bath Royal United Hospital*: Mr. E. Lewis (chairman), Miss Mason (matron). *Birmingham General Hospital*: Miss Musson, R.R.C. (matron). *Birmingham, Selly Oak Infirmary*: Miss Bodley (matron). *Brighton, Royal Sussex County Hospital*: Captain T. D. Butler, Mr. R. Ball Dodson (chairman). *Brighton Poor-Law Infirmary*: Miss Myles (matron). *Cambridge, Addenbrooke's Hospital*: Miss Crookenden, R.R.C. (matron). *Cardiff, King Edward VII.'s Hospital*: Miss E. A. M. Wilson, R.R.C. (matron). *Cheltenham General Hospital*: Miss C. Falconer (matron). *Chester Royal Infirmary*: Miss Blayney (lady superintendent). *Colchester, Essex County Hospital*: Miss W. M. Bickham (matron). *Coulsdon, Lacy Green*: Miss Watt. *Coventry and Warwickshire Hospital*: Mr. Fowler Webb, Miss Hutchinson (matron). *Derbyshire Royal Infirmary*: Miss Sutcliffe (matron). *Hamlet Union, Yorks*: Miss E. Pickersgill. *Leamington, Warneford Hospital*: Miss Jackson (matron). *Leeds General Infirmary*: Miss E. A. Innes, R.R.C. (matron). *Leicester Royal Infirmary*: Mr. H. Johnson (governor and secretary), Miss Vincent, R.R.C. (matron). *Liverpool Royal Infirmary*: Miss Cummins (lady superintendent and matron). *Liverpool Royal Southern Hospital*: Miss Bagnall (acting matron). *Manchester Royal Infirmary*: Mr. F. J. Hazell (secretary), Miss Sparshott, R.R.C. (matron). *Ancoats Hospital*: Miss Lucy Beard (matron). *Nottingham, Bagthorpe Infirmary*: Miss Dwight (matron), A. J. Blayney. *Oxford, Radcliffe Infirmary*: Miss Watt, R.R.C. (matron). *Rochester, St. Bartholomew's Hospital*: Miss Pole Hunt (matron). *Salford Union Infirmary (Hope Hospital)*: Miss Ross (matron). *Salisbury General Infirmary*: Miss Allen. *Seaford Union Infirmary*: Mr. J. Dudgeon Giles (medical superintendent). *Stoke-on-Trent, North Staffs Infirmary*: Major B. Rhodes, R.A.M.C., Miss MacMaster (matron). *Tynemouth Union Hospital*: Miss A. Flick, J. Newbiggin. *West Didsbury, Withington Hospital*: Miss Smith. *Wigan, Royal Albert Edward Infirmary*: Miss Mackintyre (matron). *Winchester, Royal Hants County Hospital*: Miss Carpenter Turner (matron). *Wolverhampton General Hospital*: Miss Hannath (matron). *Worcester General Infirmary*: Miss Herbert (matron).

Nursing Associations and Institutions.

*British Red Cross Headquarters, Boulogne*: Miss Norah Fletcher, R.R.C. *National Poor-Law Officers' Association*: Mr. T. Percival. *Nightingale Fund*: Mr. W. Bonham-Carter (secretary). *North-Western Poor-Law Conference*: Mr. R. H. Leach. *Nurses' Co-operation*: Miss Harrison. *Queen's Nurses, Northampton*: Miss Lunn (superintendent). *Queen Victoria Jubilee Nurses' Institute*: Miss Hughes, Miss Paget. *Royal British Nurses' Association*: Mr. H. Paterson, F.R.C.S., Dr. Bezly Thorne, Mrs. Campbell Thomson, Miss Macdonald. *St. Bartholomew's Hospital Nurses' League*: Miss Curtis. *Surrey Nursing Home, Surbiton*: Miss Holford.

Training Schools, Scotland.

*Edinburgh Royal Infirmary*: Professor James Ritchie, Miss Gill, R.R.C. (matron), Miss Kerr. *Glasgow Royal Infirmary*: Dr. J. Maxtone Thom (superintendent), Miss Melrose, R.R.C. (matron). *Glasgow Western Infirmary*: Miss H. Gregory Smith, R.R.C. (matron).

Training School, Ireland.

*Belfast Royal Victoria Hospital*: Miss Manser.

---

The Hon. Arthur Stanley, M.P.: Ladies and gentlemen,—I think that the first, and very pleasant, duty which we have to perform is most sincerely to thank the Treasurer and Governors of St. Thomas's Hospital for their kindness in lending us this room to-day. (Applause.) If this scheme is to come to anything, which I devoutly trust and believe it will, it will have started under very good auspices in the home of the Florence Nightingale School. I cannot help hoping that as the outcome of the Crimean War was the foundation of the magnificent system of nursing which now exists in England and her Dominions beyond the seas, so the outcome of this great and terrible war which is now going on may be a further consolidation of that profession. I want to say just a word of apology for the fact that the first list of the Council that was sent out was not complete, and also I am told by Miss Swift that some of the copies were rather illegible. Those two things were due to this reason—that we had to act in rather a hurry, and as it was on a Saturday afternoon most of our staff were away, and we were very anxious that the notices should be got out as soon as possible. The notice, even as it was, I am afraid was very short, but the attendance here to-day shows that you have been kind enough in most cases to forgive our shortcomings in that respect, and to attend here in order to learn more about the scheme for the College of Nursing.

How the College of Nursing Originated.

Now I had better begin at the beginning, which sounds a natural thing to do, and is not in this case a very formidable one, because the beginning does not date back very long. One lady wrote to me and said that she really could not have anything to do with this, because the whole origin was shrouded in mystery. Well, the one most mysterious thing about this College of Nursing, as far as it has gone, is that there has been no "mystery" about it at all: it seems to have grown up almost of itself: the idea was launched, and it has taken a more or less definite shape as it has progressed. If you will forgive me, I will go, in very few words, into what actually started the whole of this idea. I was one day discussing with Miss Swift, our Matron-in-Chief at Pall Mall, the very varying standards of certificates that were given for what I may call the amateur work in connection with nursing. In some places the examinations for home-nursing certificates and first-aid certificates are very carefully conducted, in other places they are a mere farce, and I was saying how necessary I thought it would be to have some kind of standardisation for those certificates. Miss Swift told me that that was a need that was felt not only in what I may call the lower degrees, but which was felt to a certain extent in the higher degrees; and she asked me to meet three or four matrons of some of the leading training schools in order to discuss the subject. Well, we had some meetings of a perfectly informal kind; I think at the first meeting there were probably about half a dozen present, and I think we had subsequently two or three meetings, and I was asked to bring forward something in the nature of a scheme. I think the only thing in the nature of a scheme that I brought forward was to propose that whatever body was formed should be called the College of Nursing. And my reason for this was that we already had definite, recognised bodies, recognised by the State, in the shape of the College of Physicians and the College of Surgeons, and it seemed to me a very proper complement—it seemed to me to be a rounding off, if I may so speak, of the whole subject, that there should be, with the Royal College of Surgeons and the Royal College of Physicians, a College of Nursing, which I hope in due time may become the Royal College of Nursing. I also proposed that because it appeared to me that the functions exercised by the Royal College of Physicians and the Royal College of Surgeons in relation to those two branches of the medical profession were almost exactly the functions which we should like the College of Nursing to perform in relation to the nursing profession.

Hospitals and Nurse-Training Schools Approached.

As a result of those discussions with these matrons, I was asked, with the help of Sir Cooper Perry, to write a letter to all the governing bodies of the various hospitals and nurse-training schools throughout the country. At the same time, that letter was sent by Miss Swift, and the other matrons with whom I had been consulting, to the matrons of these respective hospitals. We wished from the very beginning that the governors and the matrons should be in as full possession as possible of what we were attempting to do. We also sent out that letter in order to find out whether there was a real and genuine feeling that some such body as this College of Nursing, by whatever name it might be called, was necessary. The answers we had to those letters were sufficient to convince us of the fact that we had got on to the right line, and that it was worth our while to pursue it.

Three Fundamental Principles Agreed to.

After that letter had gone round and been considered we received representations from the various societies formed to advocate State Registration, that in their opinion they ought to be consulted, and we had two or three Conferences with representatives of those societies. I need not go at length into all the discussions that took place; I will only say for myself, speaking as one who has come quite newly into this subject, they were extremely interesting. It ended, at the last Conference, in practical agreement amongst those present that the three fundamental principles on which any association such as the proposed College of Nursing must work were these: (1) State Registration; (2) a uniform curriculum; and (3) what was called, for the sake of shortness and simplicity, the one portal—one standardised examination for entrance into the profession. Now those were discussed with the Council of the College of Nursing, and I have no hesitation in saying that we unanimously adopt those three cardinal principles as three of the objects which the College is formed to promote.

The Foundation and Establishment of the College.

Well, now, while these negotiations, or rather discussions, were proceeding, we thought it advisable to proceed at the same time with the formal requisitions which were necessary in the foundation and establishment of a body such as this. We had before us the alternative of waiting until we could get a Bill to give statutory powers to this College, or of going to the Board of Trade to have it registered as an ordinary society. I

think there was a previous attempt to have a society of this kind registered without the word "Limited" after the name. It was our original intention to proceed in that way, but I was informed on very good authority that the Board of Trade now practically refused to grant that concession, because in many cases it had been very much abused. It seemed to us that, in comparison with the magnitude of the question which we had to face, it was not very material whether the College of Nursing had the word "Limited" after it or not. (Hear, hear.) If the College of Nursing is to be the success which I think it is going to be, then the mere fact of having the word "Limited" after its name is not going to hinder its progress. Moreover, as I say—I have got no reason for saying it, but one may express a pious hope that we shall not be very long "The College of Nursing, Limited," but that we shall very soon be "The Royal College of Nursing." (Applause.)

### The Constitution of the College.

Now we had to consider, of course, the constitution of this College. We had many precedents to consider, and the one which we finally chose was that which I believe is the one of the British Medical Association. It is one which I may say I found to work very fairly well with the British Red Cross Society, and it is practically the same as that which a gentleman who was high up in the councils of the British Association told me had been found to work equally well in their case. We have a Council of thirty members, and of course we have taken power in our articles of association to increase or diminish that number if found desirable. That Council is to be elected. In order to secure certain uniformity of action during the first few months of its existence none of the members of that Council need retire until 1918, but beginning from 1918 a third of the members of that Council will retire every year. The re-election of those members, or the election of new members to fill their places, will depend entirely upon the votes of the members of the College. In other words, after 1918 there are no nominated members on that Council of any kind. We have aimed at making this a purely democratic body, a body consisting of certificated nurses, governed by certificated nurses, and in the interests of certificated nurses; and everything that can be done with that object we have done, and have put into the articles of association. (Applause.)

### The Whole Nursing Profession Represented in the Management.

But now, it is quite obvious that a Council of thirty members cannot claim to represent the whole of the nursing profession in the various parts of the United Kingdom, including Ireland. It must be a very much larger body. On the other hand, as the governing body you must have a comparatively small body, whom you are able to assemble without great difficulty, and who can really thrash out the questions in a way in which a very large body cannot do. We therefore decided to have a Consultative Board, and the real object of the meeting this afternoon is to ask your assistance in forming that Consultative Board in such a way as to make it thoroughly representative of all branches of the profession in all parts of Great Britain and Ireland. I shall ask you, when you return, to lay our views before the governing bodies and the nurses of your respective hospitals and training schools. I shall then ask you, if they agree in principle with us, to appoint from each one of those hospitals and training schools a representative to meet us here in, say, a month's time. One must give plenty of time, because some hospitals only have monthly meetings. As I say, I should ask you to appoint a representative to meet us again to arrange definitely as to how that Consultative Board shall be formed, and to give those representatives powers, if necessary, to come on to the Consultative Board themselves. We may possibly find that if, as I hope, all the training schools and other bodies connected with this subject come into the scheme, that Consultative Board is unwieldy. We may have to arrange to have representation on that Board by districts. But until we know how the scheme is taken in the country it is impossible for us to give any definite information on that subject.

### Working Committees: their Formation and Duties.

Well, now, the Council having been formed, and the Consultative Board having also been formed, there arises a necessity for working committees, committees formed of people who are interested in the definite subject for which the committee has been formed, who will, of course, carry on the spade-work, so to speak. It has been suggested by Sir Cooper Perry—and I should like to take this opportunity of saying that we are under a great debt of gratitude to Sir Cooper, not only for the work he has already done, but for his very great kindness in undertaking temporarily the position of hon. secretary—(applause)—he has suggested that to start with there shall be five committees. The first and most necessary one is a committee to deal with finance. Well, I may be asked some questions about the finance—where the money is coming from. During the past eighteen months I have had a certain amount of experience in the way of getting money. I am bound to say that practically, at the British Red Cross Society, we have not had to ask for money; money has been forthcoming. I should not like to say how I propose to get this money, but I do feel certain of this, that the British public, who have come forward so magnificently in order to ensure that the sick and wounded sailors and soldiers shall have every comfort that money can give, are not going to fail the nursing profession, which has done such gallant work with those sick and wounded. (Applause.) Then the second committee is Establishment and General Purposes—that is for what, for want of a better word, may be called the housekeeping work of the College. Thirdly, there is the Consultative Board work—that is, work in connection with the questions that will come up before the Consultative Board for consideration; all the big questions of principle will have to come up before the Consultative Board, and it is obviously necessary to have a small committee to prepare that work for the proper consideration of the Board. Then, fourthly, there is the Examination Board work, and the same thing holds good in that case. Fifthly, there is work on the Register of the College. We shall proceed at once to form a committee which will recommend to the Council what existing certificated nurses have a claim to be placed at once on the Register of the College. We hope, as I have said before, to work for a uniform curriculum, and for a definite examination or examinations which will entitle those who pass those examinations to be members of the College; but it is quite clear that you cannot ask an existing certificated nurse to go through another examination. Personally, I should be very sorry to have to go through any examination—(laughter)—and I think we may look upon those who hold certificates as having done quite enough in that line. We shall, therefore, have to form a Register, and—for I do not quite know how many years: three or four years—those who hold certificates of training schools or other certificates recognised by the Council will have the right to put their names at once upon the

Register of the College. I would say here that that Register may or not be the Register that we shall eventually take to Parliament for statutory approval. Personally, I hope it will be, but there are others who hold different views.

### The Work of the College to be Educational.

I do not wish to weary you, and therefore I can say in one sentence that the work of the College is meant from the beginning to be educational. We want to do everything that we possibly can to encourage a uniform curriculum and to get a uniform standard of examinations. We want, in other words, to do everything in our power to promote the advancement of the nursing profession in every possible way. (Hear, hear.) Our idea at this moment is that as the movement grows it will be necessary to establish centres throughout the country, and I hope that we may be able to proceed at once with the formation of boards in Scotland and Ireland. Anybody who has had anything to do with Scottish people and with the Irish know that they prefer to be managed by their own folk—(hear, hear)—and not to look to London for guidance. I have learnt that by the experience of the past eighteen months, and I propose to use the experience I have gained in order to save me from a good many difficulties that I have had in the past. We shall, of course, have our headquarters in London, and I hope, and have reason to think that my hope may come true, that among the memorials of this great war one of the greatest, one of the most permanent, will be a building worthy of the College of Nursing in a central position in London. (Applause.)

### State Recognition or Registration.

Now, of course, we invite criticism, and we are here to discuss this scheme. I have heard two objections, and two main objections, raised to it. The first one is that it will postpone the State registration of nurses; the second is that this is not the right time to bring forward a proposal of this kind, when so many nurses are away on duty at the Front and elsewhere. Well, in answer to the first, I would only say that I came into this business with an entirely open mind, and not only with an open mind, but with an entirely ignorant mind. I very soon discovered that there was no question about it, that the overwhelming feeling of the nurses, in whatever grade of the profession they were, was in favour of State recognition—I prefer to call it State recognition because I think it is a broader term—that State recognition undoubtedly implies State registration, and I have therefore been constrained, if one may use such a word—because in all these matters I have only followed—I have been constrained to put State registration as the first of the three fundamental principles upon which the College is founded. (Applause.)

### The Present is the Moment to go Ahead.

With regard to the second objection, that this is not the right time, with that I and those who have been acting with me profoundly disagree. If you do not get the nursing profession organised now, if you do not get a Bill through Parliament now, then I think we may despair of ever doing it at all. It is no use waiting until after the war. After the war people's attention will be diverted from the nursing profession in many other directions. At this moment it is not an exaggeration to say that after the actual actions of the Armies in the field, the greatest attention, the greatest interest, the greatest sympathy of the nation are directed to the nursing profession. (Applause.) We have also this danger, which is a very real one—that there will be many difficulties to be faced by the certificated nurses when the war comes to an end. There will be many hundreds of them who are now serving abroad who will come home and for whom places will have to be found. I do not pretend that this College of Nursing can find those places, but I do contend that it will be a great help to those nurses to find a strongly organised body representing nursing opinion at home when they come back. There is also the danger that a large number of women—I almost hesitate to mention the letters V.A.D. —a large number of women will come back with a very considerable amount of training; they will, many of them, have had months of work in a hospital—well, not training, I will not say training, but they will have had months of work in a hospital, and I do not think for one single minute that they are a very serious menace to the real nursing profession; but still there are a good many of them, and they will have to be reckoned with. It is just as well, I think, for the certificated nurses, before these women come back, to have got the reins of power into their own hands.

Now, ladies and gentlemen, I must thank you for the kindness with which you have listened to me. I have heard it stated that we ought, at an earlier stage, to have taken the various training schools and societies into our confidence. We felt that we could not do it until we had got the machinery set up. We felt that this was the earliest possible opportunity when it would be right to ask ladies and gentlemen to come to London, many of them from distant parts of the country, at this time, when travelling by railway is not always very easy or comfortable—we felt that it would not be right to ask them to come here until we had something definite to lay before them.

### Very Many Large Training Schools Support the College.

I shall be very glad to answer any questions. I have tried, as briefly as possible, to lay before you what our scheme is. I am glad to say that we have got the approval and support of a very large number of the great training schools. That is evidenced, I think, by the first list of the Council which we have been able to send out to you, and in connection with that list there is one remark I ought to make, which I am very pleased to make. It will probably have been noticed that the name of a representative of the London Hospital does not occur on that list, but I have been asked to say that that does not necessarily mean that they are holding out against the scheme, or are opposed to it. We all know and deplore the unfortunate accident that has happened to Lord Knutsford, the chairman of that hospital. We know that by that accident he is precluded from taking any active part in the work of the hospital, I fear for several months to come, but I had a talk with him only two days before his accident; we went thoroughly into the whole subject of this College, and if we can meet the views of the London Hospital on two or three points we, I believe, shall obtain their support. It would be very incomplete if we had a scheme like this with the London Hospital and all those larger number of nurses connected with it left out—(hear, hear)—and I am glad to say that in the actual points at issue between us I personally found myself entirely in sympathy with the views expressed by Lord Knutsford, and, from what I know of the opinions of those who have been advising me, and those now on the Council, I believe that they will be in agreement with his views also. I am still not without hope that we may go forward as a united body. We have already appointed a small committee to confer with representatives of those bodies who have been supporting State registration. Personally I believe there is practically no difference in our opinions. All that has happened is that we have somewhat changed the method of procedure. Instead of waiting for Parliament to set up the machinery, we have set up the machinery, which we could perfectly well do without Parliament's help, and we shall go to Parliament for sanction for that machinery when we find it necessary to do so. Ladies and gentlemen, I must thank you again for having listened to me so kindly. I feel perfectly certain that when the history of this war comes to be written, one of the finest chapters in that history will deal with the work of nurses during the war. (Hear, hear.) I want, and all those who are working with me want, to set up in this country an administration for the nursing profession that shall be worthy of the magnificent service that the nurses themselves have rendered to the sick and wounded and to the country during this present war. (Applause.)

### THE DISCUSSION.

Mr. T. Hall-Hall (for the Treasurer of St. Thomas's Hospital) : Mr. Chairman, my Lords, ladies, and gentlemen,—The Treasurer of this hospital is, most unfortunately, not well enough this afternoon to come here, as he had hoped to do, to personally welcome you as host to this place. And unfortunately Mr. Minett, who is not only the senior member of the Almoners' Committee of the hospital, but is also Chairman of the Nightingale Fund, has been called away from London. The Treasurer has therefore asked me to welcome you here in his name, and to express his regret that he cannot be here himself, and his great pleasure in having been able to place the Governors' Hall at your disposal for your discussion this afternoon. When the scheme of a Nurses' College was first brought forward, the Treasurer brought that before the Governors, and they gave it their warm approval as a general idea. They felt that the establishment of such a College as that which was proposed in Mr. Stanley's first letter would be of immense service to the nurses and of great value in advancing not only the position of the nurses themselves, but of patients throughout the country. Further than that this hospital has not at present been able to go, and it must be well understood that, although the meeting is taking place within the walls of St. Thomas's Hospital, the governing body of that institution have as yet not expressed any opinion on the merits of the scheme or on the principles which the proposed College is to adopt. The College has now been constituted, and the first Council has been formed. As soon as the Council has definitely formulated its principles, we hope that they will be laid before the Governors of this hospital, and that we shall have an opportunity of discussing them and considering how far we can give them our support. At present this hospital wants it to be fully understood that we retain an entirely free hand. That being the case, I do not propose, nor am I a competent person to do it, to enter into any discussion on the merits of the matter to-day, and all I have got to do is to perorate. They say an orator should always have a good peroration, and I have rather a good one. I have got it written down here. The Treasurer has asked me to say that tea will be provided in the Nightingale Room at the close of the meeting. (Laughter and "Hear, hear.")

The Chairman : Might I suggest that any lady or gentleman wishing to address the meeting will come up to the platform? After what has been said I should like to add that nobody need be in any way compromised by being at this meeting this afternoon. This is only a meeting to discuss how we can best form a Consultative Board that will be representative of the nursing profession throughout the country.

### The Representation of Ireland.

Miss Manser (Royal Victoria Hospital, Belfast) : Mr. Chairman, I have been asked to point out that on the list of names of the Council sent round to the various institutions Ireland is not represented at all. I feel rather diffident in mentioning this after our Chairman's remarks, but still at the same time, remembering that he has told us Ireland likes to govern herself—though there is a great difference of opinion about that—we should like to be represented on the Council, and we should like, if possible, both North and South to be represented. I think that is all I wanted to say, Mr. Chairman.

The Chairman : May I say, in answer to that, that we have allocated two places on the Council to Ireland? There has been a meeting of a Committee appointed by the Royal Colleges of Surgeons and Physicians in Ireland —they only met a few days ago, and I only got their letter yesterday, to the effect that they thought the nurses of Ireland would be unanimously in support of the scheme if they could have a fifth of the whole governing body. (Laughter.) Well, we had got so far that we could not possibly give them a fifth of the governing body. But I have a sort of feeling that Ireland will get what she wants. (Laughter.) At all events, we have kept specially two places for Ireland, in the same way that two places were kept for Scotland, which have now been filled.

Miss Manser : Thank you. That is just what we wanted.

### An Amusing Speech.

Dr. Fisher (Shoreditch Infirmary) : I think, Sir, that all nurses and all doctors must have great sympathy with your project, and I think the feeling has been, amongst teachers, for a long time that it was much to be deplored that State Registration had not come. The College that you propose to set up—or, rather, that you have already set up—meets with my very warm approval, and I speak, I think, as a representative of other superintendents of infirmaries. I approve of a uniform curriculum, a uniform period of training, one portal, and registration. But where I feel disquiet, Sir, is in regard to the machinery which has been set up. You make this statement in your preliminary letter to us—that "just as the Royal Colleges of Physicians and Surgeons, through the Conjoint Board, organise the teaching and examination of medical students; as the Chartered Institutes of Accountants, Surveyors, Engineers, and other bodies, as barristers and solicitors organise the teaching and examining of candidates for entrance to their respective professions, so do I feel most strongly that now is the right time for some such movement in nurse training." But, Sir, the nursing profession is an anomaly. Doctors teach doctors, lawyers lawyers, and engineers engineers; but nurses are taught by medical men—("No, no," and laughter)—and the teaching they receive is just sufficient, in the teachers' opinion, to enable them to rationally carry out the directions and treatment that the doctor proposes to give. Therefore I think such a responsible body as the Council of this College should have a very large and preponderating number of its representatives appointed from among leading members of the medical profession. ("No, no.") Oh, I have every sympathy with nurses. (Laughter.) But doctors alone, I think, with their intimate knowledge of the patients, know exactly how much nurses should know, and along what lines they should be trained. Too much knowledge, to a nurse, is dangerous. (Laughter.) If they wish to be doctors, that portal is open to them. You have set up your machinery, as you said, Sir, and you have stated that you propose that this machinery shall be composed to the extent of two-thirds of matrons and nurses. But you do not mention how many medical men you wish to have on this Council, or how many medical members of the Council you propose to appoint. The relations between medical men and nurses should always be of the most amiable nature. (Laughter.) We do not want to be faced with a position where the nurse has a profession and the medical man has a profession, and they are at variance. I think superintendents of infirmaries are in a very happy position to speak in this direction, because we spend many years in very large institutions with large numbers of beds, running up to a thousand; we control large numbers of nurses, and we know what responsibilities we have, and we know exactly what the needs of the nurses are, and we know what to expect from the superintendent nurses and the matrons. The matrons have to guide the nurses to a large extent, but the matrons look practically—I may say almost entirely—to the medical teachers for the instruction of these nurses. Such instruction as the matron is capable of giving she receives from the medical teachers. (Laughter.) Well, Sir, my remarks, I am glad to find, have been heard to the end of the room, and they have evidently caused much merriment; but this is the time of all times when such opinions should be expressed, and, although I dislike public speaking intensely, I have taken advantage of your courteous invitation by coming up on to the platform and expressing my views. (Applause.)

The Chairman: I think perhaps it will be convenient if I just take up any points made by the speakers, if I can, without waiting till the end.

Lord Sandhurst: As I understand, Sir, you suggest that the Council, or one of these bodies, is to be elected, but I did not gather in what form the election was to be made.

### How Council Elected in 1918.

The Chairman: In answer to Lord Sandhurst and Dr. Fisher, that was a point I was going to raise. The two-thirds matrons or nurses practising their profession only applies to the first nominated Council. We nominated only fifteen, which was the minimum number we could nominate. The Council may be thirty, and we nominated fifteen. Of those, ten had to be matrons or nurses; in other words, two-thirds had to be matrons or nurses. That is all over now. After those fifteen were nominated to form the first Council they co-opted a few other members, and will co-opt up to the number, I imagine, of thirty. That Council will remain in power till 1918. At the end of that time one-third retires every year. We have provided for a postal ballot of the nurses. Every member of the College will receive a voting paper which will enable her to vote either to re-elect the retiring members or to elect others in their place. And I may say now that all those papers will be secret, and therefore it will be impossible for us to tell how the votes are cast, or, rather, impossible for us to tell who casts each vote; we shall only know the result when all the returns are in.

### The V.A.D.s and the College.

Mr. Bonham-Carter: I ought, perhaps, as Secretary of the Nightingale Fund, to put in the same caveat as was put in by Mr. Hall-Hall. As you all know, both St. Thomas's Hospital and the Nightingale Fund, when this question of State Registration came before the world over twenty years ago, took a strong line against it. They have not yet agreed to decide upon approval of the form this College is going to take. When the original letter was written we approved of what was originally proposed, and gave it our blessing. But we have not met since to discuss the matter, and therefore I do not propose to express any settled views to-day, although I may say I am myself personally in favour of some College being established. The only thing I wanted to understand a little bit more was, how the College would propose to deal with these ladies of whom we speak here with bated breath, the ladies who bear the mystic letters "V.A.D." after their names. Because, I take it, one of the first things to be said with regard to any scheme of registration is that, whereas you are going to provide entirely for the registered nurse, and, I suppose, to take power to prosecute anyone holding herself out to be a registered nurse without being one, there will be a very large number of excellent women who will have had some knowledge of nursing—we must not say "training" in nursing, but knowledge of nursing—which can be made use of, as we have seen in this war; these women will number, I believe, many thousands, and the College will have in some way or other to deal with them. If the Chairman will kindly give us some little explanation as to how it is proposed that these women shall be dealt with, I shall then be able to bring that question before my committee, amongst other matters which we shall have to consider.

The Chairman: The answer to that is that I hope the College will have in its own hands the examination and granting of certificates as to all classes of women's work in hospitals. As you know, at present members of Voluntary Aid Detachments have, before they can become members of a Detachment, to get their home-nursing certificate and first-aid certificate. I hope—of course, I have no right to speak for the V.A.D.s—but I hope that it will be part of the work of the College of Nursing to conduct those examinations, to see that they are properly conducted, and to give their certificates; in other words, instead of getting certificates of very varying value, as they do now, the fact that they hold the certificate of the College of Nursing would show that at all events a certain standard had been reached.

### Questions for the Consultative Board to Determine.

Mr. Lewis (Royal United Hospital, Bath): There are just one or two points which I would like explained. I do not speak with "bated breath" of the V.A.D.s. I have had a good deal to do with them during the last eighteen months, and I know a good deal about them. I have been for very many years strongly in favour of State Registration, and I am glad to know that one of the first objects of the proposed College is State Registration. I look upon the nursing profession as a very important one, and it is one which has not been properly recognised in the same way as the medical profession, dentistry, and other professions have been. I take the view that in many cases the nurse is quite as important to the patient as the doctor—(hear, hear)—and I think that those women who have had three years' training in a hospital, and have had practical experi-

### SOME QUESTIONS FOR THE CONSULTATIVE BOARD.

ence of nursing, and have been taught by experienced doctors and matrons, should be in some degree safeguarded from those women who, to my own knowledge and in my own experience, go for a year or two into a hospital but never get a certificate, and who go out into the country and practise as nurses with no proper certificate whatever.

What I want to get at is with regard to the question of the last speaker, as to the V.A.D. nurses. All honour to the work they have done during the war. But the time has now arrived, I think, for State Registration to protect those women who have worked to obtain a certificate, because when the war is over we shall be flooded with a large number of women who have done excellent work, who have a smattering of nursing, and who perhaps, as regards nursing wounded soldiers, are very capable, but who have no practical experience of ordinary hospital work. And unless this registration is hurried forward before the war is over, so as to enable us to register those who have proper qualifications, we shall be flooded with a lot of partially trained women who will be practising as nurses. Therefore I hope the intention is that State Registration is to be pushed forward rather before the College itself.

There is one question I want cleared up. Of course I know the standard varies, but I want to know how the College intends to deal with the examinations. In the case of the hospital which I represent, our examination is fairly stringent by our medical men and matrons. Is it the intention of the College to do away with certificates granted by hospitals in that way? Do all have to go to the College for a certificate, or are certificates to be granted by other institutions in a similar way as at the present time?

Then there is another thing. You have mentioned, Sir, that the Register of Nurses must be formed now, immediately the College is started. What standard do you intend to take with regard to those nurses registered? Some give three, and some four years. What is to be the standard?

You also mentioned in your speech that you took it for granted that probably Parliament would not take the Register of the College of Nursing as the basis of State Registration. I do not see why that should be so, or why there should be more stringent rules with regard to nurses than there are in the case of midwives and dentists. Those nurses and dentists who had already obtained qualifications did not require to have new ones, and it seems to me that those nurses who have obtained proper qualifications in the past should be included in this Register by Parliament. I rather gathered from your remarks that you did not take that view.

Then you mentioned also the London Hospital. I hope the London Hospital may come into this scheme, but as I understand their training is for two years, and from many years' experience of hospital work I do not think two years is enough. I should be sorry to see the standard lowered to two years, even for the sake of getting in the London Hospital. (Loud applause.) The London Hospital may be a very important hospital, and Lord Knutsford, as we all know, has taken a lot of interest in hospitals generally; but three years' training, I think, is the minimum period that ought to be required before granting a certificate for nursing.

Then I want to know how the College proposes to deal with the training of nurses in the future, after the College is started—whether they intend to recognise certain training schools. I think the standard of 100 beds is generally recognised. I should like to know whether the College is going to adopt that standard, or to set up another standard for themselves.

Mr. Frankau (St. George's Hospital): My question is this, How far the College will treat the report of the training school, hospital, or whatever it may be, as a condition precedent to admission to an examination? I believe that in most of the professions there is some sort of evidence required from another body, not the examining body, that a person is a fit and proper person to be admitted as a member of that profession. Well, I am quite sure, from what little I know of hospital work, that what is learned in the hospital as to a nurse's capacity, *morale*, qualifications, and other things can only be known to that hospital, and no examining body in the world can find out those things in the short time devoted to an examination.

What I should like to get clear is this. I can speak for my own hospital, and we are certainly in sympathy with, and inclined to weigh, this proposition to the best of our ability, but I feel sure that until we have something tangible, not so much as to the constitution of this body, but as to how it is going to work its rules—perhaps I am asking a rather unfair question in asking to be told what is going to happen, but I feel that until we have that sort of fact before us it is most difficult for an ordinary layman to make up his mind whether to support this or not. We certainly put a value on our own certificate. Speaking for myself, I do not care so much about our own certificate, but I do feel that in the interest of the public and in the interest of the nurses the report of the individual hospital should have great weight, even if it be not made an actual condition precedent to a nurse being allowed to go in for this statutory examination. Perhaps the Chairman can tell me what is intended with regard to this matter.

The Chairman: These questions which have been raised by the last two speakers are exactly the questions, really, that will have to be dealt with at first by the Consultative Board which we are asking you to form. (Hear, hear.) I do not know—nobody knows—on what terms present certificated nurses will be allowed on to this Register which we propose to form at once. The whole object of getting this Consultative Board is that we may be able to consult the representatives of the nursing profession on that particular point. We want to know what their views are as to who should be on this Register. We want to know what their views are as to what shall be the conditions three years hence, say, for argument's sake, when we are in full working force—what shall be the conditions precedent to allowing nurses to come on to the Register. I hold very strongly with Mr. Frankau that the report of the hospital itself in which the nurse has been trained is of the very greatest possible value. (Hear, hear.) I hold equally that nobody can possibly tell by a mere examination whether a woman is qualified to be a certificated nurse or not. We shall have to combine the two. But the way in which that is to be done is exactly what we are asking your advice about. If you will form for the College a really strong, representative Consultative Board, by which these questions can be thrashed out, we shall have taken an enormous stride towards the consolidating of the nursing profession. We shall be doing it then with the knowledge that we are doing it on the lines laid down by the experience and the knowledge of the nurses themselves.

As regards Mr Lewis's first question, about registration being hurried forward, I feel so strongly about it that I believe that if, in conjunction with the Societies for State Registration, we could only get an agreed Bill—

and I do not see any insuperable objection—I should certainly be inclined to urge it forward and to see whether it would not be possible to get the Government to take it up as a matter of war urgency. It is, of course, quite impossible to get through a private Bill at this time unless the Government take it up. If we go with anything like united opinion on the part of nurses, I believe at this moment we could get it through.

Then as regards examinations—whether they are to come from the College—if we grow to be the body I hope we shall, I think examinations will have to be taken something on the lines of the Oxford and Cambridge Examinations, and be taken all over the country. I was talking to a gentleman only this morning who is intimately connected with that organisation, and he explained to me how it was worked; and I do not think we should have any difficulty in working on the same lines. The nurses would not have to come to the College; the College would go to them.

Mr. Lewis has not clearly understood me in regard to one matter. He said that I suggested Parliament would not take this register that we are to form. That is not the intention I meant to convey. If that is the register that the nursing profession generally wish us to take to Parliament, of course we will take it. Some people have expressed the view that they do not think that the register of a voluntary body such as this is the one to which statutory powers should be given, but personally, being connected with the College, I think it is the one to which statutory powers should be given. But I purposely made the remark which I did in order to leave that question open. At all events, there is one thing which is quite clear. Parliament is much more likely to give you statutory recognition of a register they have in front of them than to give you powers to make one. We are much more likely to get it through in that way.

### Some Passing Matters.

Miss Bodley (Selly Oak, Birmingham Union): Mr. Chairman, I believe I am the only representative of the largest Union in England, and I should like to ask that there may be representation of our Union on the Consultative Board. I believe that the clerk has already written to you, Sir, and that he received no invitation to to-day's meeting. And also the chief medical officer of our Union has had no invitation. I believe I am right in saying so. We have over 3,000 beds for the sick, and we train over 250 nurses in the year. I was talking to Mr. Curtis yesterday, and he feels that he would like to know a little more about the scheme, because he has not been able to consider it properly yet.

The Chairman: I am sorry the invitation has not reached your Union, and I think there must have been a mistake somewhere. I hope Miss Bodley will make it clear that it was a mistake and not an intentional omission.

Miss Bodley: Then we may hope that a representative will be allowed to be sent up from our Union.

The Chairman: Certainly.

Mr. Tom Percival (vice-president, Poor-Law Officers' Association): May I explain that I have received quite a number of similar inquiries? I think the trouble arises from the fact that the invitations were sent to medical superintendents and matrons, instead of to the clerks to the Guardians.

Mr. Foster (London Hospital): Mr. Chairman,—May I ask whether it is possible to leave a place upon the Council for Lord Knutsford—because unfortunately he will not be able to do any work for some months—and that Mr. Morris may meanwhile represent him?

The Chairman: Well, I have to say the Council is very nearly full now. From my old friendship with Lord Knutsford, and because of the help he has given me during the war, there is nobody whom personally I should welcome upon the Board more heartily than Lord Knutsford. But, of course, it is for the Council themselves to say who should fill the vacancies. I cannot promise that they will leave one open, but I will promise to do all I can to get the help and support of Lord Knutsford.

Mr. Foster: Thank you.

### Professor Ritchie of Edinburgh.

Professor Ritchie (Edinburgh Royal Infirmary): Mr. Chairman, I happen to be in the unfortunate position of representing one of the outlying parts of the kingdom to which you have referred as liking to manage their own affairs. But with regard to this particular subject I do not think I can say that that is so. Not only the Edinburgh Infirmary, but the nursing schools of Scotland generally, are certainly at one with the main object of this College—namely, the State registration of nurses—and I do not think there will be any more loyal supporters of State registration than the representatives of the Scottish hospitals. (Hear, hear.)

With regard to the formation of a Consultative Board, which I take it is the object of this meeting—or making arrangements for the formation of a Consultative Board—there is only one question which I should like to put to you. When the Edinburgh Infirmary representatives go back to the Board, as they will next week, with a report of this meeting, we are certain to be asked regarding the constitution of the College of Nursing, and therefore I would ask if the Council proposes to circulate as soon as possible copies of the memorandum and articles of association to the hospitals in order that they may be considered? I am certain that we recognise that many of the questions which have been asked this afternoon are questions which can only be settled after the College is thoroughly in working order, and questions which really come under the consideration of the Council of the College. But I am perfectly certain—speaking not only for myself, but for others—that the lay members of hospital boards throughout the country would almost certainly want to know what the exact constitution of the College was before they proceeded to elect representatives to attend this meeting, which, as you say, is to be held in about a month's time.

Perhaps there is one other point I might put forward. Some names have been mentioned for the Council. I think it is perhaps rather difficult to mention names at this meeting, because it is not one of the functions of the meeting to do so, as I understand. But as names have been mentioned I should like very strongly to bring under the notice of the Council the name of Dr. Mackintosh, of the Western Infirmary, Glasgow, as a man who would, I am sure, be of the utmost value on that body. As everyone knows, he is one of the greatest authorities upon hospital construction and upon all matters relating to the superintendentship of hospitals, and therefore I would put forward his name for the earnest consideration of the Council.

The Chairman: I shall be very glad to put the name of Dr. Mackintosh before the Council at their next meeting. I assure Professor Ritchie that if we do not elect him it will only be because there is no room. I think we possibly have to extend the Council somewhat, but we must wait for that until we see how representative we can get the Consultative Board.

### The Memorandum of Association.

As regards circulating copies of the memorandum and articles of association, we shall be very glad to send them to anyone who writes for them. But I would only make this remark. When you are going to form an Association such as this, you go to the Board of Trade for a memorandum and articles of association, and it is the custom, as any gentleman who has had anything to do with business knows, to include in that memorandum and in the articles every possible thing which you can think of. Sir Charles Russell, than whom I should think there is nobody cleverer in this particular matter, told me that in this memorandum and articles of association we had included practically everything that was possible and everything that was not; he said that probably the only things we had omitted were powers to open a restaurant and to build a Dreadnought. (Laughter.) It is necessary to take full and absolute powers to do everything you can possibly want to do, and I hope those who read the memorandum and articles of association will bear this fact in mind, and remember that we have tried to think of every possible emergency and contingency and to take powers accordingly. If anybody will kindly write for the memorandum and articles of association we shall have much pleasure in sending them.

Mr. Horton Smith, K.C. (University College Hospital): I am Vice-Chairman of the University College Hospital, and I should like to give the names of our matron, Miss Dora Finch, and of Captain Butler, who is one of our committee, as members of the Consultative Board which you speak of.

The Chairman: I must just say one word. Mr. Horton Smith has been kind enough to propose these names for the Consultative Board. As regards one of them, I think we have gone one better, because we have asked Captain Butler to be one of the first Vice-Presidents of the College, and I am glad to say he has been kind enough to accept. (Hear, hear.)

### Some Published Registers of Nurses.

Sir Henry Burdett: I thought, as we have so large a gathering here, representing so many parts of the country and so many institutions, that I might properly remind the Council and those present that registration has made great strides in the larger hospitals, and that some of the best managed hospitals throughout the kingdom already have their own registers, and those registers are very complete; they contain the names of all the nurses who have been certificated from the hospitals, together with their subsequent careers. And I cannot help thinking that if every hospital which has adopted this plan would send to Mr. Stanley a copy of its register, the latest edition it has, with particulars about it, it would help the preliminary arrangements and much of the detail work that has to be done in order to give the College the best possible start. The register with which I am familiar is most excellently and carefully prepared, and contains an immense amount of information.

### The Royal British Nurses' Association.

Dr. Bezly Thorne: I rise to plead for the Royal British Nurses' Association. As probably most of you are aware, that Association was founded some years ago, and has been favoured by a Royal Charter. The grantees of the Royal Charter were fifteen ladies and fifteen doctors. The fifteen ladies consist of a Royal Princess and fourteen trained nurses. That body has now for some years, under the powers—very wide powers—granted it by Royal Charter, been the only independent examining body in the United Kingdom which examines nurses for three years' training and exercises the power of granting diplomas in nursing. Now, Sir, I do not quite know in what capacity my friend who is sitting over to your right acts on your Council, but I wish here to take this opportunity of entering a plea that the Royal British Nurses' Association should be represented on your Council by a nurse. There are 3,000 enrolled members on our Register, and, as I have already stated, we do perform already the unique function of issuing diplomas to nurses on the basis of an independent examination. And I think it would only be just and right that we should have on your Council, among your representatives, a trained nurse.

The Chairman: I shall certainly put that recommendation of Dr. Bezley Thorne's in front of the Council. The Royal British Nurses' Association have been most friendly to us throughout the proceedings, and we should certainly wish to do anything that is their desire. I do not know, but I expect that among those matrons and nurses who are on our Council there are certain to be some members of the Royal British Nurses' Association. But I will have that looked into.

### A Matron's Confidential Register of Nurses.

Miss Musson (Birmingham): I should like to remove an impression which I think will do a great deal of harm if it goes out into the nursing world. I for one have not the slightest intention of giving my confidential hospital register to any committee whatever. If this is to be a self-governing body of nurses it can only admit individual nurses who apply for admission. I am quite sure we should, none of us, ever dream of giving our confidential hospital registers, or even a list of certificated nurses, to any body without their authority. I should like to make that quite clear.

The Chairman: I should equally like to make it clear to Miss Musson that I did not ask for it. (Laughter.)

### The Next Meeting of Hospital Representatives.

Mr. Garratt (Royal Free Hospital): I should like to ask whether from this time henceforth all the proceedings of the College of Nursing are to be done in the name of the Council, and whether all future communications sent out to the hospitals and other institutions asking for representatives will be sent in the name of the Council. I might mention also that there appears to be a certain amount of feeling that a longer period than a month should elapse in which the names of representatives for the Consultative Board should be obtained. Many hospitals do not meet—perhaps, if they meet now, they do not meet again for another month, and there would not be time to appoint representatives, and I think it might be well if instructions were given that the Council, who, I presume, will summon the next meeting, should fix it for a time which will enable a longer period than a month to be given in which to send in the names of representatives.

There was another suggestion also made, and that was that in order that the Consultative Board might not be of an unwieldy size, it might be possible for it to be more or less representative of districts—that the matter might be decentralised, so that districts, more or less, might nominate the representatives for the Consultative Board.

The Chairman: May I just answer those points, taking the last one first? I think it may very likely be found advisable to get representation by districts, but until we know who are coming in it is difficult to say. Mr. Comyns Berkeley has been kind enough to give me just a sketch of the organisation of the British Medical Association, and it is just possible that something similar may be found to meet our needs.

Then as regards letters going out in the name of the Council, I most heartily agree to that. You will have nothing more over my name. (Laughter.) Now that the Council has been appointed I shall be only too glad to abdicate in their favour. I only had to do it at first because I happened to be the chairman, for the moment, of a big neutral body that had no axe to grind, and that had no particular history in relation to this movement, and therefore it was suggested that I should do it. But I am only too glad to hand that work on to somebody else.

Then as to whether longer notice than a month should be given for nominations to the Consultative Board. Of course, I do not want to rush it, but, on the other hand, we want to get ahead with this work as quickly as possible. We do not know how long the war will last—I think it will last longer than a month, but I do not know how long. I may remind the meeting that there is no definite number fixed for the Consultative Board, and there would not be a closed door even at the end of the month. We should rather like to adhere to the time of one month if we could.

Sir Henry Burdett: There could be a special meeting, Sir; the Committees can arrange that.

The Chairman: Yes. I hope they might think it of sufficient importance to have a special meeting.

Mr. Garratt: Do I understand that notice will be sent to the hospitals—a definite notice, inviting them to appoint representatives?

The Chairman: Yes.

Mr. Garratt: And it would be a month from the date of the notice?

The Chairman: Yes, put it like that; that would give a few days extra. While we are on this subject I should like to say this: I hope we may be fortunate enough to get the loan of this room again; we could not have a better place in which to meet. But is Friday a convenient day, and is three o'clock a convenient hour? Those who think that Friday at three would be convenient kindly hold up their hands. And those who do not? Then I think the only vote we have is a unanimous one.

### Lancashire Guardians and the College of Nursing.

Mr. Leach (representing the North-Western Poor-Law Conference, Lancashire): I take it, Mr. Chairman, that the College is already formed, and that this meeting this afternoon is convened for the purpose of advising the Council on the formation of a Consultative Board. Am I right in that, Sir?

The Chairman: Yes, that is right.

Mr. Leach: Well, I have been sent up from the North-Western District of Lancashire and Cheshire to represent Boards of Guardians in those two counties who themselves are the governors of quite a number of training schools. For instance, in Lancashire, in the large training schools of Manchester, Liverpool, and other large Poor-Law centres, there must be under training now, and it is an increasing number, somewhere about 1,200 or 1,400 nurses or more. Therefore any college, association, or body which can assist the Poor-Law authorities in securing the adequate training of nurses should be welcomed by those authorities. Three years ago the North-Western Conference adopted a scheme for the training of nurses in order to standardise the training, because every school was giving its own training and its own certificate, and there was a feeling that all the certificates were not of equal merit, and that to get a standardised curriculum and examination would secure an equal value to all the certificates. I understand that is one of the main planks of this College.

Now my position, Sir, is this. It seems to me, from what one had heard, that whoever takes part in the formation, or assists in the formation, of a Consultative Board must be in full agreement with the articles of association of this College. I am not here to say that the North-Western Conference is in disagreement with any article of association or in agreement with any article of association, because at the present time we do not know anything about it; and I have come on to the platform to suggest this—that between now and your next meeting we may have sent to us a copy of the articles of association, and also that a copy of the articles of association should be sent to the Secretary of the Poor-Law Unions Association, which is a body existing under statute, so that these articles may be fully considered by their executives, and that those in agreement with the articles of association should have the privilege of sending a representative to the Consultative Board. I simply make that request—that in sending out invitations for representatives on this Board you will not forget the Poor-Law Unions Association, particularly the North-Western Poor-Law hospitals. It must be remembered that there is no body in existence that is collectively under greater responsibility for the sick poor than Boards of Guardians in the aggregate are. According to the annual report of the Local Government Board, our nurses and nurses in training in our Poor-Law hospitals are not less than 10,000 in number, and when you remember that large number of nurses you may easily understand what a huge number of patients have to be dealt with. (Applause.)

The Chairman: Well, ladies and gentlemen, Mr. Leach speaks for the North-Western Poor-Law Conference, representing Lancashire and Cheshire. I come from Lancashire myself, and I am perfectly certain I should have a very poor time there if I did not pay proper attention to any representations from that quarter. (Laughter.) I shall be very glad to bring that before the Consultative Committee, and I am very glad to know that you take such a kindly interest in the College.

Now I do not see anyone else getting up, and therefore I think, as you are all busy people, this might be considered the end of what I, personally, thank you for as a very happy and interesting meeting.

### Lord Sandhurst Gives Sympathetic Support.

Lord Sandhurst: Ladies and gentlemen, I hope you will allow me to move a vote of thanks to Mr. Stanley for his conduct in the chair. (Hear, hear.) I have been to a great many meetings, and I never knew one better conducted, especially on a subject which we know has many difficulties surrounding it. And I was very sorry to hear that there was a chance of Mr. Stanley abdicating, as he called it, his present place in regard to the College, because from his experience, and from the experience we have had of him in the chair this afternoon, I am quite certain he would have been most helpful in steering this project through difficulties and making a good job of what is obviously a very big job—namely, the setting firmly on its feet of this College.

*See page* vi *for remainder.*

