# A Correction

**Published:** 1916-04-22

**Authors:** J. Dudgeon Giles

**Affiliations:** Medical Superintendent. Salford Union Infirmary, Pendleton, Manchester


*To the Editor of* THE HOSPITAL.

*To the Editor of* THE HOSPITAL.

### A Correction.

*To the Editor of* THE HOSPITAL.

SIR,—May I point out a misprint on page 63 of the current number of THE HOSPITAL (April 15), twenty-fourth line from bottom of right-hand column, where *Sea*ford Union Infirmary appears instead of *Sal*ford?—Yours faithfully, J. DUDGEON GILES,
Medical Superintendent.

Salford Union Infirmary, Pendleton, Manchester,
April 16, 1916.

[We are indebted to Dr. Dudgeon Giles for this correction. In the information which reached us on which the article was based, Seaford, not Salford, Union Infirmary was given; hence the error.—ED. THE HOSPITAL.]

